# Comparison of Patient Satisfaction in Inpatient Care Provided by Hospitalists and Nonhospitalists in South Korea

**DOI:** 10.3390/ijerph18158101

**Published:** 2021-07-30

**Authors:** Wonjeong Chae, Juyeong Kim, Eun-Cheol Park, Sung-In Jang

**Affiliations:** 1 BK21 FOUR R&E Center for Precision Public Health, College of Health Science, Korea University, Seoul 02841, Korea; wjchae0816@korea.ac.kr; 2 Institute of Health Services Research, Yonsei University, Seoul 03722, Korea; kjy394@syu.ac.kr (J.K.); ecpark@yuhs.ac (E.-C.P.); 3 Department of Health & Human Performance, Sahmyook University, Seoul 03722, Korea; 4 Department of Preventive Medicine, College of Medicine, Yonsei University, Seoul 03722, Korea

**Keywords:** hospitalist, patient experience, patient satisfaction, inpatient care, quality of care

## Abstract

Background: A Korean hospitalist is a medical doctor in charge of inpatient care during hospital stays. The purpose of this study is to examine the patient satisfaction of hospitalist patients compared to non-hospitalist patients. Patient satisfaction is closely related to the outcome, quality, safety, and cost of care. Thus, seeking to achieve high patient satisfaction is essential in the inpatient care setting. Design, setting, and participants: This is a case-control study based on patient satisfaction survey by the Korean Health Insurance Review and Assessment Service. We measured patients’ satisfaction in physician accessibility, consultation and care service skills, and overall satisfaction through logistic regression analyses. A total of 3871 patients from 18 facilities responded to 18 questionnaires and had health insurance claim data. Results: Hospitalist patients presented higher satisfaction during the hospital stay compared to non-hospitalist patients. For example, as per accessibility, hospitalist patients could meet their attending physician more than twice a day (OR: 3.46, 95% CI: 2.82–4.24). Concerning consultation and care service skills, hospitalists’ explanations on the condition and care plans were easy to understand (OR: 2.33, 95% CI: 1.89–2.88). Moreover, overall satisfaction was significantly higher (β: 0.431, *p* < 0.0001). Subgroup analyses were conducted by medical division and region. Hospitalist patients in the surgical department and the rural area had greater patient satisfaction in all aspects of the survey than non-hospitalist patients. Conclusions: Hospitalists’ patients showed higher satisfaction during the hospital stay. Our study discovered that hospitalists could provide high-quality care as they provide onsite care continuously from admission to discharge.

## 1. Introduction

Patients’ satisfaction and experience during their hospital stay is one factor that reflects the care quality of a hospital [1,2,3,4,5]. Patient satisfaction and experience are often reviewed by government bodies and accreditation agencies to grade hospitals. In addition, patient satisfaction has direct and indirect influence in the outcome, quality, safety, and cost of care [5,6,7,8,9,10]. Therefore, hospitals are seeking ways to improve patient satisfaction and experience.

Several factors have been found to enhance patients’ satisfaction in the hospital, including not only the doctors’ precise medical performance, but also their communication and interpersonal skills [2,5,11,12]. These skills were shown to be strong among primary care physicians (PCPs) who can build better relationships with patients over several years [12,13,14,15,16,17]. However, for inpatient care, some studies indicate that hospitalists, i.e., medical doctors who are in charge of inpatient care during hospital stays [18], provide inpatient care more efficiently than PCPs and non-hospitalists [11,15,19]. In addition, better care quality, patient safety, and outcomes from hospitalists have been shown to lead to higher patient satisfaction during the hospital stay [9,19,20,21,22]. Studies and interest in patient satisfaction have increased in recent years for the indicator of health quality [23,24,25,26,27,28,29]. The patient’s experience and satisfaction, which are the feedback from the patients, could be measured in the aspects of communication, professional practice, responsiveness to patients, and environmental factors [24,25,26,27,28,29,30,31]. The previous studies discussed limitations to the applied intervention and investigated the degree of effectiveness of patient-centered quality care. However, those feedbacks from the patient would contribute to providing essential evidence to improve patient care quality [25,26,27,28,31].

In 2016, South Korea implemented a Korean hospitalist system [32,33]. Prior to this implementation, most inpatient care had been provided by medical residents. The hospitalist model was implemented institutionally by the government to suit the current Korean health care system [32,33,34]. The criteria that define Korean hospitalists are: (1) medical specialist in internal medicine or surgery, (2) provide medical service to hospitalist ward inpatients only, and (3) stationed near the hospitalist ward. Those requirements are established to implement Korean hospitalists suitable for the Korean healthcare system, especially in the new medicine discipline’s beginning stage and system [33]. Hospitalists in Korea provide care only to patients admitted to the hospitalist ward due to the fee schedule. As Korea is under the National Health Insurance system, a specific fee schedule is created for the hospitalists [33]. Such criteria of Korean hospitalists allow improving accessibility, patient safety, and patient satisfaction during hospital stays. As a result, hospitalists only manage inpatient care and are medical specialists from internal medicine and surgery divisions. Due to the fee schedule structure for hospitalists, this new system was implemented by unit, with 50 beds per ward [35]. The implementation of this system was led by low patient satisfaction of care and patient demands for medical specialty care during their hospital stay. Thus, we hypothesized that patient satisfaction would increase with the hospitalist system.

Since Wachter and Goldman first introduced the term in 1996, hospitalist care has been compared with non-hospitalist care in several studies on various aspects such as patient’s safety, patient satisfaction, care outcome, and care quality [8,9,12,18,19,20,36,37,38]. Today, more countries are employing the hospitalist model for their inpatient care and management [21,39,40]. Other Asian countries such as Taiwan, Singapore, and Japan have adopted the hospitalist model and have improved their inpatient care outcomes [21,40]. Thus, we expected that the new Korean hospitalist system would also improve outcomes, including patient satisfaction.

In this study, we surveyed patients who were admitted to hospitalist and non-hospitalist wards to compare patient satisfaction by measuring the attending physician’s accessibility, consultation and care service skills, and overall satisfaction. Therefore, our study aimed to investigate patient’s satisfaction with hospitalist care during the hospital stays as one aspect of evaluating the Korean Hospitalist System after its implementation.

## 2. Methods

### 2.1. Data, Study Design, and Study Population

Data was collected from the hospitals that implemented the Korea hospitalist system in 2016 through the Health Insurance Review and Assessment Service (HIRA) which is the government agency. A total of 18 facilities administered a standardized patient survey in hospitalist wards as case groups, and non-hospitalist wards as control groups from September 2016 to December 2016. Those 18 facilities were all facilities where the implementation of the Korean Hospitalist System initially during the government pilot study was voluntary [33]. This study was conducted to evaluate the hospitalist system cooperative with the government agency. With the hospitalist group as the case and the non-hospitalist group as the control group, we conducted a quasi-experimental study.

The survey was administered by nurses before patients were discharged from the hospital, and the answers provided data related to patient satisfaction and experience. Additional data related to the patient’s clinical conditions were extracted from the discharge summary sheet written by the attending physician and HIRA insurance claim data.

**Figure 1:**
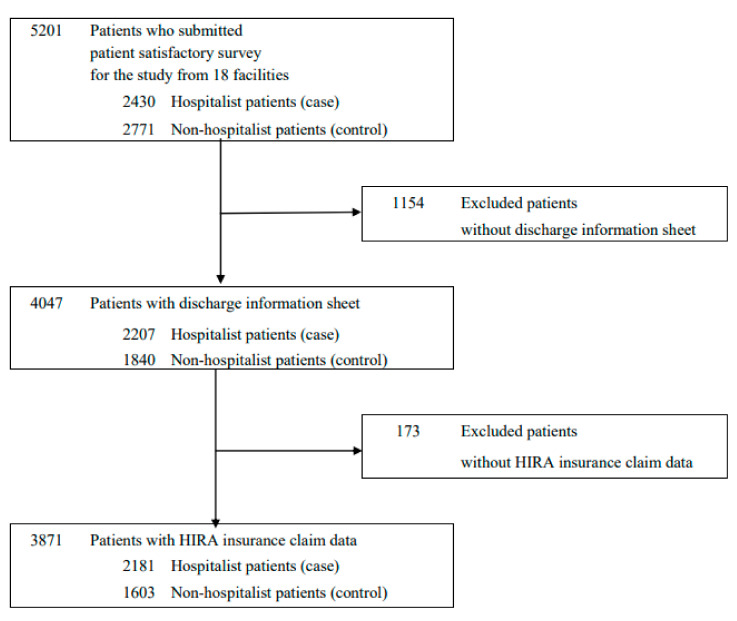
Flowchart of study population selection.

A total of 5201 patients responded to the survey. However, we eliminated individuals who did not have a discharge summary sheet and who were unable to be matched with the HIRA database (Figure 1). In order for the HIRA to retrieve data from the hospital’s claim, it would take some days to be processed within their system. Thus, we could not include in the study claim data of patients admitted toward the end of the study period that could not fully be generated by the HIRA. Therefore, our final study population included 3784 patients: 2181 hospitalist patients and 1603 non-hospitalist patients.

### 2.2. Patient Satisfaction Survey and Discharge Summary Sheet

The survey was distributed and collected by the HIRA under the Korean Ministry of Health and Welfare’s supervision. All patients and facility information were de-identified for the study. To develop the survey, we reviewed previous studies on healthcare assessment tools and quality evaluation criteria from the Tool to Assess Inpatient Satisfaction with Care from Hospitalists and the Hospital Consumer Assessment of Healthcare Providers and System [38,41,42]. The survey was distributed by nurses before the discharge.

A discharge information summary sheet was developed to review the comorbidity and severity of the patients. Patient satisfaction and experience could differ depending on their health condition. Therefore, the discharge information summary contained the patient’s clinical conditions and severity. These indicators were developed after consulting with medical experts.

### 2.3. Measure of Patient Satisfaction

Patient satisfaction was the outcome of this study. Patients answered questions on 3 domains with 18–25 questions per domain. These domains were accessibility, consultation and service skills, and overall satisfaction. Patients responded by using common rating scales—either by selecting a response (strongly dissatisfied, dissatisfied, average, satisfied, or strongly satisfied) or rating between 0 and 10 (most satisfied) [14,41,42]. We used “strongly satisfied” as the outcome for the logistic regression that those who chose other responses were grouped into one group. The supplementary analyses were conducted by using “strongly satisfied” and “satisfied” as the outcome.

### 2.4. Confounding Variables

A variable that could have an association with an independent variable and a dependent variable is called a confounding variable [43]. In the study, controlling the confounding variable is necessary to investigate the association between the independent and dependent variables. A series of potential confounding variables were identified to adjust for multivariable analysis. These variables include sex, age, medical division, admission type, and region. In addition, we adjusted for patient severity and comorbidity during hospital stay, the data for which were extracted from the discharge summary sheet and HIRA insurance claim data. These variables included surgery, general anesthesia, intensive care unit (ICU) transfer, death, hypertension, diabetes, hepatitis, tuberculosis, dialysis, Charlson’s comorbidity index (CCI) score, and the attending physician’s subjective score on the severity level of the patient.

### 2.5. Statistical Analysis

For general characteristics of the study population, the chi-square test and t-test were performed to compare differences between groups. The multicollinearity in the model was tested using the variance inflation factors (VIFs). VIFs were less than 10, indicating that there were no excessive correlations between variables in the statistical model [44]. To determine the association between the attending physician type and patient satisfaction, we constructed a fully adjusted multivariable logistic regression model. For overall satisfaction, we applied linear regression analyses. The results were considered significant at a *p*-value lower than 0.05. All analyses were performed using SAS version 9.4 (SAS Institute, Cary, NC, USA).

### 2.6. Ethical Statement

The data were obtained as part of the policy evaluation by the Health Insurance Review and Assessment Service. This study used secondary data without the patient’s information that was exempted from Yonsei University Health System, Severance Hospital Institutional Review Board.

## 3. Results

### 3.1. General Characteristics and Health Condition of the Study Population

The general characteristics of the patients are presented in Table 1. Among all patients, 57.6% (*n* = 2181) were treated by hospitalists and 42.4% (*n* = 1603) were treated by non-hospitalists. By age group, 16.6% (*n* = 861) of patients were aged 50–59 years, 16.7% (*n* = 868) aged 60–69 years, and 15.2% (*n* = 791) aged 70–79 years. By region, 70.5% (*n* = 2667) of patients were admitted in capital area hospitals. Regarding the length of hospital stay, hospitalist patients stayed an average of 6.50 days and non-hospitalist patients stayed an average of 5.69 days. Out of 251 transferred patients, 57.8% (*n* = 145) patients were in the hospitalist wards. As per death case during hospital stays, there were 19 death and 63.2% (*n* = 12) occurred in the hospitalist wards. Regarding clinical conditions, 61.7% patients had hypertension and diabetes, 66.9% had hepatitis, 61.4% had tuberculosis, and 57.3% had dialysis. Concerning comorbidity, hospitalists had more patients with a high CCI score than non-hospitalists. According to the attending physician’s subjective severity score, hospitalists responded with higher severity than non-hospitalists.

### 3.2. Patient Satisfaction in Hospitalist Care Compared to Non-Hospitalist Care

Table 2 shows the association between the type of attending physician and patient satisfaction for patients who responded “strongly satisfied”. Compared with non-hospitalist patients, hospitalist patients showed greater satisfaction while staying in the hospital. The analyses were conducted on each survey question and its response. In the hospitalist wards, patients were able to meet the attending physician soon after admission (odds ratio (OR), 2.57; 95% confidence interval (CI), 1.99–3.33), have a consultation with the attending physician after requesting one (OR, 2.75; 95% CI, 2.09–3.61), and meet with the attending physician more than twice a day (OR, 3.46; 95% CI, 2.82–4.24). Regarding the attending physician’s consultation and care service skills, hospitalists’ patients reported higher scores than non-hospitalist patients. Hospitalists were easy to understand (OR, 2.33; 95% CI, 1.89–2.88) and showed interest in the patients’ views and opinion about their health (OR, 2.25; 95% CI, 1.83–2.77). Lastly, overall satisfaction was significantly higher in hospitalist patients than in non-hospitalist patients (*p*-value: <0.0001). The results of the analysis on satisfied and strongly satisfied are presented in Supplementary Table S1. Statistical results with *p*-value are presented in Supplementary Table S2.

### 3.3. Patient Satisfaction in Hospitalist Ward Stratified by the Medical Division

Subgroup analyses were conducted by medical division and region. Hospitalist patients who were admitted in the internal medicine or surgery departments showed higher patient satisfaction than non-hospitalist patients (Table 3). The analyses were conducted on each survey question and its response. Accessibility to their attending physician was three times higher in hospitalist patients than in non-hospitalist patients (internal medicine: OR, 3.47; 95% CI, 2.73–4.41; surgery: OR, 3.68; 95% CI, 2.43–5.55). Statistical results with *p*-value are presented in supplementary Table S3.

### 3.4. Patient Satisfaction in Hospitalist Ward Stratified by Region

Table 4 shows the results of the analysis by region. The analyses were conducted on each survey question and its response. Rural area patients expressed greater satisfaction in accessibility, including their ability to meet their attending physician soon after admission (OR, 42.40; 95% CI, 8.84–203.25), have a consultation when they requested one (OR, 51.51; 95% CI, 13.58–195.35), and spend an adequate amount of time in consultation (OR, 83.43; 95% CI, 9.93–701.11) than capital area patients.

## 4. Discussion

This case-control study compared patient satisfaction between patients who received care from hospitalists or non-hospitalists at 18 Korean facilities. Consistent with our hypothesis, our analysis revealed that patients who received care from hospitalists had higher satisfaction and better experience during their hospital stay than those who received care from non-hospitalists. Patients reported significantly greater satisfaction with hospitalists with respect to accessibility, consultation and care skills, and overall satisfaction. From the study, patients who were cared for by hospitalists had higher satisfaction. In addition, patients were experiencing quicker responses from the attending physician hospitalist as they were able to meet their doctor more than twice a day, able to meet when they requested, and able to meet soon after the admission. Moreover, the hospitalist explained better the patient’s condition, procedure, and care process. Our results are similar to the previous study indicating that patients who received hospitalist care had higher satisfaction especially in communication skills [14,45].

The results of subgroup analysis on the medical division and regions allowed us to have valuable insight into the Korean health system and its needs. The objective of the subgroup on medical divisions was not to compare internal medicine and surgery departments directly. Instead of the direct comparison between two groups, the analyses were conducted to understand how hospitalist care impacted patient satisfaction. As the results show that patients cared for by hospitalists in both departments had much higher satisfaction, we were able to see the need for a hospitalist system implementation. Additionally, hospitalists with a specialty in surgery are a unique element of the Korean hospitalist system that other countries could consider adopting and expand to the surgery department model. The results from subgroup analysis on regions also support the needs of hospitalist care for inpatient care. The lack of health resources in the rural area led to difficulty in healthcare access, especially during hospital stays. However, through implementing the hospitalist system, patients saw doctors and received care from specialists from admission to discharge. Therefore, we expect improvements in medical outcomes, patient safety, and care quality as the system evolves.

High patient satisfaction indicates that patients have received high quality care [4,5,10]. Reviewing patients’ satisfaction could be a tool to measure care quality, especially for patients who received inpatient care [12,14,17,46]. Since the term was first introduced in 1996, the role of the hospitalist has evolved [18,32,36]. Hospitalists manage the high volume of inpatients more efficiently and provide high-quality care while reducing costs and length of stay [11,22,46,47,48]. In addition, patients who received care from hospitalists reported equal or higher satisfaction and experience during their hospital stay [6,7,14,38]. Higher patient satisfaction can be achieved via the doctor’s medical knowledge, treatment skills, and patient-centered care, all of which leads to building rapport and trust with patients [6,8,46,49].

Previous studies have demonstrated that patients prefer PCPs and have higher satisfaction with PCPs [16,17]. Because they have built a relationship over several years, patients experienced a continuum of care with their PCPs [12,16,17]. By contrast, other studies have reported higher patient satisfaction with hospitalists for various measures, especially overall satisfaction and discharge planning [19,20,40]. Similarly, our findings also showed higher satisfaction with hospitalists for these two measures. Moreover, concerning the attending physician’s consultation and care service skills, hospitalist patients reported higher scores for diagnosing and treating skills and communication skills than non-hospitalist patients. These results also indicate that hospitalist patients experienced greater satisfaction than non-hospitalist patients.

In order to improve the quality of care, patient feedback such as patient satisfaction during hospital stays was often measured [24,25,26,27,29,30,31]. Patient feedback provides the information that can lead to provide patient-centered care [24,27]. For example, communication is an essential factor that influences patients’ feedback. The patients appreciate how the medical staff responded to their request and carries the conversation [26,28,29]. Patients could have different experiences during hospital stays that the physician’s interpersonal skills, communication skills, and explanation with genuine courtesy affect the patients’ satisfaction and the feedback [24,28]. The physician’s professional skill is essential to patient care quality that directly impacts the patient’s feedback [26,28,50]. Related to the professional skills, the physician’s response to the patient also influences the quality of care and health outcome that can be measured in patient feedbacks [26,27,51,52,53]. Our study revealed the higher satisfaction among hospitalist’s patients in communication skills, interpersonal skills, professionalism, and accessibility. Thus, we believe hospitalists would contribute significantly to improving care quality in inpatient care management.

Similar to other countries that implemented the hospitalist model, Korea improved patient satisfaction with hospitalists [8,21,40]. High satisfaction and experience are also associated with high-quality care and patient safety [4,9]. Prior to the hospitalist system, the majority of inpatient care was provided by medical residents in Korea. Therefore, the factors that increase patient satisfaction were low with those residents [32,54,55]. Instead, patients expressed demands of inpatient care from specialists [32,34,35,54]. Patients were not able to meet their attending physicians during their hospital stay, and residents were not able to fulfill the needs of their patients. However, when hospitalists were onsite with the patient 24/7 with easy access to specialists, patients felt that they were receiving patient-centered care that could increase care quality and patient safety [10,14,22]. Thus, patients have shown higher satisfaction and experience with hospitalist care. Furthermore, patients were willing to pay extra costs to receive care from specialists and hospitalists during their hospital stay.

### Strengths and Limitations

There are several limitations to our study. First, it was a cross-sectional study, such that the measure of exposure and outcome occurs simultaneously. Therefore, we could not test for the causality of the exposure and the outcome [56]. To overcome this limitation, we adjusted the analysis for various potential confounders, such as demographic data, clinical conditions during hospital stay, and severity of health conditions. However, it is possible that we did not include all possible confounders, such as the patient’s medical history and comorbidities. The patients chose where to be admitted between the hospitalist ward and the traditional care ward. Thus, a patient’s choice could lead to bias in the study on top of the patient’s health condition. Moreover, the cost of care could influence the patient’s satisfaction with the provided care that we suggest including in a future study. Second, the implementation of the hospitalist system was fairly new at the time of study, thus it could have had an indirect influence on patient satisfaction. Most facilities included in the study were tertiary educational hospitals, a bias that may have also affected our outcome. However, we controlled for this variability by ensuring that each facility had a case group and a control group to reduce biases by hospital type. Third, this study was conducted to evaluate the newly implement healthcare system in Korea, the Korean Hospitalist System in from the patient’s perspective. Thus, it is a quasi-experimental study to evaluate the implemented system. The study followed the case-control design with a case group of hospitalist ward patients and a control group of non-hospitalist ward patients. For future studies after the mid-phase of implementation of the system, we suggest analyzing with extending data collection and a study design that meets statistical requirements of the case-control design. Fourth, we measured patient satisfaction and experience using a survey that we developed to evaluate the new system. The survey was carefully developed after reviewing previous studies evaluating patient satisfaction and experience during hospital stays. However, the validity of the survey was not assessed, thus it may not be a representative tool to measure overall patient satisfaction. Moreover, the data collection and survey conducted rely on the practical process to conduct in aspects of the newly implemented pilot study evaluation for the health policy rather than research-driven solid studies. Fifth, the study period was relatively short and we suggest that a follow-up study be conducted when the system will be more widely implemented in Korea.

Nevertheless, our study contributes important findings about hospitalists and inpatient care services. Firstly, this is the first study on hospitalists and their inpatient care in Korea. Therefore, data were collected from all the hospitals where the new system had been implemented at the time of the study. Because the Korean healthcare system is a single insurer model, we were able to eliminate confounders from various insurance types. Secondly, we collected data from both hospitalist and non-hospitalist patients at the same facility. This allowed us to employ a case-control study design using real data from patients. Thirdly, we provided evidence that hospitalists deliver high-quality care with high patient satisfaction. Concerns about care quality, patient satisfaction, and experience between hospitalists and non-hospitalists are still ongoing. However, our results demonstrate positive outcomes from hospitalists’ inpatient care and can be used to advocate for expanding the hospitalist system. 

## 5. Conclusions

In conclusion, we found that patients cared for by hospitalists show improved satisfaction and experience during their hospital stay. This result indicates that hospitalists were able to provide high quality care as they are providing onsite care continuously from admission to discharge. Thus, we recommend that policymakers and hospital leaders expand the hospitalist model for inpatients care.

## Figures and Tables

**Table 1:** General characteristics of the study population.

		Total		Hospitalist Patients		Non-Hospitalist Patients		χ^2 †^
		3784	%	2181	%	1603	%	<0.0001
**Sex**								0.8302
	Male	1843	48.7	1059	57.5	784	42.5	
	Female	1941	51.3	1122	57.8	819	42.2	
**Age**								0.1363
	≥19	37	1	21	56.8	16	43.2	
	20–29	128	3.4	62	48.4	66	51.6	
	30–39	262	6.9	151	57.6	111	42.4	
	40–49	528	14	286	54.2	242	45.8	
	50–59	861	22.8	487	56.6	374	43.4	
	60–69	868	22.9	495	57	373	43	
	70–79	791	20.9	484	61.2	307	38.8	
	≥80	309	8.2	195	63.1	114	36.9	
**Admitted department**								<0.0001
	Internal Medicine	2706	71.5	1644	60.8	1062	39.2	
	Surgery	1078	28.5	537	49.8	541	50.2	
**Admission type**								0.0057
	General admission	3330	88	1892	56.8	1438	43.2	
	ER admission ^‡^	454	12	289	63.7	165	36.3	
**Admission experience**								0.1886
	Yes	3019	79.8	1714	56.8	1305	43.2	
	No	719	19	430	59.8	289	40.2	
**Region**								<0.0001
	Capital	2667	70.5	1379	51.7	1288	48.3	
	Rural	1117	29.5	802	71.8	315	28.2	
**Length of Stay** **(Mean, SD ^†^)**		6.16 (5.79)		6.50 (6.12)		5.69 (5.34)		0.0116
**Surgery**		3722		2138		1584		0.0152
	Yes	952	25.6	515	54.1	437	45.9	
	No	2770	74.4	1623	58.6	1147	41.4	
**General anesthesia**		3717		2136		1581		0.0763
	Yes	903	24.3	496	54.9	407	45.1	
	No	2814	75.7	1640	58.3	1174	41.7	
**ICU transfer**		3714		2134		1580		<0.0001
	Yes	251	6.8	145	57.8	106	42.2	
	No	3463	93.2	1989	57.4	1474	42.6	
**Death**		3713		2134		1579		0.6154
	Yes	19	0.5	12	63.2	7	36.8	
	No	3694	99.5	2122	57.4	1572	42.6	
**Hypertension**		3752		2158		1594		0.0001
	Yes	1328	35.4	819	61.7	509	38.3	
	No	2424	64.6	1339	55.2	1085	44.8	
**Diabetes**		3746		2155		1591		0.0105
	Yes	741	19.8	457	61.7	284	38.3	
	No	3005	80.2	1698	56.5	1307	43.5	
**Hepatitis**		3738		2146		1592		0.0156
	Yes	151	4.0	101	66.9	50	33.1	
	No	3587	96.0	2045	57.0	1542	43.0	
**Tuberculosis**		3739		2147		1592		0.3104
	Yes	145	3.9	89	61.4	56	38.6	
	No	3594	96.1	2058	57.3	1536	42.7	
**Dialysis**		3735		2146		1589		0.8477
	Yes	75	2.0	43	57.3	32	42.7	
	No	3660	98.0	2103	57.5	1557	42.5	
**Charlson’s Comorbidity Index**								<0.0001
	0	1075	28.4	623	58	452	42	
	1	393	10.4	229	58.3	164	41.7	
	2	1614	42.7	916	56.8	698	43.2	
	3+	702	18.6	413	58.8	289	41.2	
AP’s subjective severity *								<0.0001
	0~9	239	6.3	99	41.4	140	58.6	
	10~19	444	11.7	226	50.9	218	49.1	
	20~29	619	16.4	341	55.1	278	44.9	
	30~39	526	13.9	276	52.5	250	47.5	
	40~49	541	14.3	301	55.6	240	44.4	
	50~59	588	15.5	435	74	153	26	
	60~69	351	9.3	265	75.5	86	24.5	
	70~79	259	6.8	144	55.6	115	44.4	
	80~89	195	5.2	78	40	117	60	
	90~100	22	0.6	16	72.7	6	27.3	

^†^ Results of univariate analysis (chi-squared test); ‡ Admitted through emergency room; * AP: attending physician; ^+^ standard deviation.

**Table 2:** Results of logistic regression on the patient satisfaction on attending physician in the hospitalist ward compared to the non-hospitalist ward ^‡^.

	Satisfaction	
	OR	95% CI
**Accessibility to attending physician**		
I was able to meet attending physician soon after the admission	2.57	(1.99–3.33)
I was able to consult with attending physician when I request a consultation	2.75	(2.09–3.61)
Attending physician has responded quickly upon the pain management request	2.23	(1.81–2.75)
Attending physician has responded quickly upon medicine and procedure request	2.25	(1.82–2.78)
I was able to meet attending physician more than twice a day (including rounding)	3.46	(2.82–4.24)
Attending physician has spent adequate amount of time in consultation, procedure, and care services	2.42	(1.88–3.12)
I was able to have answer to my question related to care during admission period of time	2.39	(1.92–2.98)
**Attending physician’s consultation and care service skills**		
Attending physician let me talk without interrupting	2.22	(1.82–2.70)
Attending physician checked to be sure I understood everything	2.08	(1.68–2.58)
Attending physician communicated fully related to my care and possible negative outcomes	1.78	(1.45–2.18)
Attending physician was not in a rush when he/she was with me	1.94	(1.55–2.42)
Attending physician’s explanation was easy to understand	2.33	(1.89–2.88)
Attending physician showed interest in my views and options about my health	2.25	(1.83–2.77)
How do you rate attending physician’s skill in diagnosing and treating your medical condition?	1.86	(1.51–2.31)
Attending physician kept me informed of the plans for my care	2.02	(1.62–2.53)
How do you rate attending physician’s fund of knowledge?	1.65	(1.34–2.02)
Attending physician effectively prepared me for discharge	1.58	(1.25–2.10)
Attending physician re-explained discharge guidelines in details at discharge	1.98	(1.58–2.49)
**Overall satisfaction evaluation**		
Overall satisfaction on attending physician (beta, *p*-value)	0.431	<0.0001
Overall satisfaction on hospital service (beta, *p*-value)	0.371	<0.0001
Overall satisfaction on my health status prior to the admission (beta, *p*-value)	0.263	0.0004
I would pay extra cost to admitted in the medical ward where care and services are provided specialist (hospitalist)	44.07	(31.69–61.29)

^‡^ Fully adjusted for the analysis (adjusted variables: sex, age, medical division, admission type, and region, surgery, general anesthesia, intensive care unit (ICU) transfer, death, hypertension, diabetes, hepatitis, tuberculosis, dialysis, Charlson’s comorbidity index (CCI) score).

**Table 3:** Results of logistic regression on the patient satisfaction on attending physician in the hospitalist ward compared to the non-hospitalist ward by the medical division ^‡^.

	Medical Division			
	Internal Medicine		Surgery	
	OR	95% CI	OR	95% CI
**Accessibility to attending physician**				
I was able to meet attending physician soon after the admission	3.38	(2.38–4.81)	1.30	(0.86–1.97)
I was able to consult with attending physician when I request a consultation	3.59	(2.48–5.20)	1.15	(0.73–1.81)
Attending physician has responded quickly upon the pain management request	3.90	(2.20–3.84)	1.06	(0.74–1.51)
Attending physician has responded quickly upon medicine and procedure request	2.84	(2.16–3.75)	1.14	(0.79–1.63)
I was able to meet attending physician more than twice a day (including rounding)	3.47	(2.73–4.41)	3.68	(2.43–5.55)
Attending physician has spent adequate amount of time in consultation, procedure, and care services	3.19	(2.26–4.50)	1.20	(0.80–1.81)
I was able to have answer to my question related to care during admission period of time	3.09	(2.31–4.13)	1.06	(0.72–1.55)
**Attending physician’s consultation and care service**				
Attending physician let me talk without interrupting	2.70	(2.10–3.47)	1.19	(0.79–1.78)
Attending physician checked to be sure I understood everything	2.53	(1.92–3.32)	1.15	(0.74–1.80)
Attending physician communicated fully related to my care and possible negative outcomes	2.01	(1.56–2.60)	0.98	(0.64–1.50)
Attending physician was not in a rush when he/she was with me	2.55	(1.89–3.43)	0.82	(0.52–1.27)
Attending physician’s explanation was easy to understand	2.86	(2.17–3.77)	1.20	(0.79–1.84)
Attending physician showed interest in my views and options about my health	2.88	(2.20–3.76)	1.55	(0.73–1.70)
How do you rate attending physician’s skill in diagnosing and treating your medical condition?	2.29	(1.72–3.03)	0.92	(0.61–1.41)
Attending physician kept me informed of the plans for my care	2.71	(2.01–3.65)	0.89	(0.57–1.38)
How do you rate attending physician’s fund of knowledge?	1.71	(1.32–2.22)	1.43	(0.93–2.19)
Attending physician effectively prepared me for discharge	1.77	(1.30–2.41)	1.08	(0.67–1.74)
Attending physician re-explained discharge guidelines in details at discharge	2.27	(1.69–3.05)	1.03	(0.65–1.62)
**Surgical patient only**				
I am satisfied with the overall treatment and management after surgery			1.02	(0.66–1.60)
I am received satisfactory care when I requested for pain control at the surgical site			1.10	(0.71–1.72)
I am satisfied with the operation site infection management			1.17	(0.75–1.82)
**Satisfaction evaluation**				
Overall satisfaction on attending physician (beta, *p*-value)	0.610	<0.0001	0.253	0.0117
Overall satisfaction on hospital service (beta, *p*-value)	0.554	<0.0001	0.138	0.2289
Overall satisfaction on my health status prior to the admission (beta, *p*-value)	0.296	<0.0001	0.293	0.0156
I would pay extra cost to admitted in the medical ward where care and services are provided specialist (hospitalist)	37.94	(23.81–60.47)	29.65	(11.24–78.22)

^‡^ Fully adjusted for the analysis (adjusted variables: sex, age, medical division, admission type, and region, surgery, general anesthesia, intensive care unit (ICU) transfer, death, hypertension, diabetes, hepatitis, tuberculosis, dialysis, Charlson’s comorbidity index (CCI) score).

**Table 4:** Results of logistic regression on the patient satisfaction on attending physician in the hospitalist ward compared to the non-hospitalist ward by region ^‡^.

	Region			
	Capital Area		Rural Area	
	OR	95% CI	OR	95% CI
**Accessibility to attending physician**				
I was able to meet attending physician soon after the admission	1.69	(1.27–2.25)	42.40	(8.84–203.25)
I was able to consult with attending physician when I request a consultation	1.50	(1.10–2.04)	51.51	(13.58–195.35)
Attending physician has responded quickly upon the pain management request	1.28	(1.01–1.63)	35.48	(12.91–97.49)
Attending physician has responded quickly upon medicine and procedure request	1.43	(1.13–1.81)	25.82	(9.81–67.98)
I was able to meet attending physician more than twice a day (including rounding)	2.08	(1.63–2.63)	30.94	(16.04–68.67)
Attending physician has spent adequate amount of time in consultation, procedure, and care services	1.56	(1.18–2.05)	83.43	(9.93–701.11)
I was able to have answer to my question related to care during admission period of time	1.29	(1.01–1.65)	49.76	(29.74–83.26)
**Attending physician’s consultation and care service**				
Attending physician let me talk without interrupting	1.57	(1.25–1.96)	6.74	(3.62–12.58)
Attending physician checked to be sure I understood everything	1.33	(1.04–1.69)	15.71	(6.80–36.29)
Attending physician communicated fully related to my care and possible negative outcomes	1.09	(0.86–1.37)	6.34	(3.45–11.68)
Attending physician was not in a rush when he/she was with me	1.19	(0.93–1.53)	16.22	(5.76–45.69)
Attending physician’s explanation was easy to understand	1.37	(1.08–1.74)	15.54	(6.53–36.99)
Attending physician showed interest in my views and options about my health	1.38	(1.09–1.75)	9.14	(4.44–18.80)
How do you rate attending physician’s skill in diagnosing and treating your medical condition?	1.27	(1.00–1.61)	13.79	(5.65–33.67)
Attending physician kept me informed of the plans for my care	1.34	(0.97–1.60)	21.17	(7.49–58.81)
How do you rate attending physician’s fund of knowledge?	1.20	(0.94–1.50)	4.19	(2.25–7.82)
Attending physician effectively prepared me for discharge	0.92	(0.70–1.21)	17.37	(6.12–49.31)
Attending physician re-explained discharge guidelines in details at discharge	1.07	(0.82–1.39)	25.63	(9.74–67.44)
**Satisfaction evaluation**				
Overall satisfaction on attending physician (beta, *p*-value)	0.247	0.0027	1.013	<0.0001
Overall satisfaction on hospital service (beta, *p*-value)	0.223	0.0099	0.748	<0.0001
Overall satisfaction on my health status prior to the admission (beta, *p*-value)	0.173	0.0583	0.503	0.0013
I would pay extra cost to admitted in the medical ward where care and services are provided specialist (hospitalist)	42.36	(27.76–6.40)	102.11	(39.10–266.67)

^‡^ Fully adjusted for the analysis (adjusted variables: sex, age, medical division, admission type, and region, surgery, general anesthesia, intensive care unit (ICU) transfer, death, hypertension, diabetes, hepatitis, tuberculosis, dialysis, Charlson’s comorbidity index (CCI) score).

## Data Availability

Restrictions apply to the availability of these data. Data was obtained from HIRA and are available with the permission of HIRA (https://opendata.hira.or.kr/home.do).

## Supplementary Materials

The following are available online at https://www.mdpi.com/article/10.3390/ijerph18158101/s1, Table S1: Results of logistic regression on the patient satisfaction on attending physician in the hospitalist ward compared to the non-hospitalist ward for agree and strongly agree; Table S2: Results of logistic regression on the patient satisfaction on attending physician in the hospitalist ward compared to the non-hospitalist ward with *p*-value; Table S3: Results of logistic regression on the patient satisfaction on attending physician in the hospitalist ward compared to the non-hospitalist ward by region with *p*-value.
