# Supplementary material for: Comparison of Patient Satisfaction in Inpatient Care Provided by Hospitalists and Nonhospitalists in South Korea
Source: Int J Environ Res Public Health. 2021 Jul 30;18(15):8101. doi: 10.3390/ijerph18158101 (PMC8345769; doi:10.3390/ijerph18158101)
Supplement: Supplementary file 1 [file ijerph-18-08101-s001.zip › ijerph-1272641-supplementary.pdf]

Supplementary Materials

Table S1. Results of logistic regression on the patient satisfaction on attending physician in the hospitalist ward compared to the non-hospitalist ward for agree and strongly agree

	Satisfaction†	
	**OR**	**95% CI**
**Accessibility to attending physician**		
I was able to meet attending physician soon after the admission	1.66	(1.37 – 2.01)
I was able to consult with attending physician when I request a consultation	2.23	(1.85 – 2.68)
Attending physician has responded quickly upon the pain management request	2.24	(1.77 – 2.84)
Attending physician has responded quickly upon medicine and procedure request	2.16	(1.70 – 2.75)
I was able to meet attending physician more than twice a day (including rounding)	3.42	(2.81 – 4.16)
Attending physician has spend adequate amount of time in consultation, procedure, and care services	2.31	(1.88 – 2.83)
I was able to have answer to my question related to care during admission period of time	2.93	(2.34 – 3.68)
**Attending physician's consultation and care service skills**		
Attending physician let me talk without interrupting	2.73	(1.96 – 3.79)
Attending physician checked to be sure I understood everything	2.54	(1.97 – 3.28)
Attending physician communicated fully related to my care and possible negative outcomes	1.59	(1.23 – 2.05)
Attending physician was not in a rush when he/she was with me	2.14	(1.73 – 2.65)
Attending physician's explanation was easy to understand	2.73	(2.13 – 3.50)
Attending physician showed interest in my views and options about my health	3.48	(2.66 – 4.56)
How do you rate attending physician's skill in diagnosing and treating your medical condition?	2.01	(1.54 – 2.61)
Attending physician kept me informed of the plans for my care	2.24	(1.79 – 2.80)
How do you rate attending physician's fund of knowledge?	2.00	(1.62 – 2.47)
Attending physician effectively prepared me for discharge	1.91	(1.54 – 2.38)
Attending physician re-explained discharge guidelines in details at discharge	2.33	(1.86 – 2.93)
**Overall satisfaction evaluation**		
Overall satisfaction on attending physician (beta, *p*-value)	0.434	<0.0001
Overall satisfaction on hospital service (beta, *p*-value)	0.375	<0.0001
Overall satisfaction on my health status prior to the admission (beta, *p*-value)	0.283	<0.0001
I would pay extra cost to admitted in the medical ward where care and services are provided specialist (hospitalist)	41.86	(30.39 – 57.66)

* Fully adjusted for the analysis; † Satisfaction measured in combination of 'agree' and 'strongly agree'

Table S2. Results of logistic regression on the patient satisfaction on attending physician in the hospitalist ward compared to the non-hospitalist ward with *p*-value ‡.

	Satisfaction	
	**OR**	***p*-value**
**Accessibility to attending physician**		
I was able to meet attending physician soon after the admission	2.57	<0.0001
I was able to consult with attending physician when I request a consultation	2.75	<0.0001
Attending physician has responded quickly upon the pain management request	2.23	<0.0001
Attending physician has responded quickly upon medicine and procedure request	2.25	<0.0001
I was able to meet attending physician more than twice a day (including rounding)	3.46	<0.0001
Attending physician has spent adequate amount of time in consultation, procedure, and care services	2.42	<0.0001
I was able to have answer to my question related to care during admission period of time	2.39	<0.0001
**Attending physician's consultation and care service skills**		
Attending physician let me talk without interrupting	2.22	<0.0001
Attending physician checked to be sure I understood everything	2.08	<0.0001
Attending physician communicated fully related to my care and possible negative outcomes	1.78	<0.0001
Attending physician was not in a rush when he/she was with me	1.94	<0.0001
Attending physician's explanation was easy to understand	2.33	<0.0001
Attending physician showed interest in my views and options about my health	2.25	<0.0001
How do you rate attending physician's skill in diagnosing and treating your medical condition?	1.86	<0.0001
Attending physician kept me informed of the plans for my care	2.02	<0.0001
How do you rate attending physician's fund of knowledge?	1.65	<0.0001
Attending physician effectively prepared me for discharge	1.58	<0.0001
Attending physician re-explained discharge guidelines in details at discharge	1.98	<0.0001
**Overall satisfaction evaluation**		
Overall satisfaction on attending physician (beta, *p*-value)	0.431	<0.0001
Overall satisfaction on hospital service (beta, *p*-value)	0.371	<0.0001
Overall satisfaction on my health status prior to the admission (beta, *p*-value)	0.263	0.0004
I would pay extra cost to admitted in the medical ward where care and services are provided specialist (hospitalist)	44.07	<0.0001

‡Fully adjusted for the analysis (adjusted variables: sex, age, medical division, admission type, and region, surgery, general anesthesia, intensive care unit (ICU) transfer, death, hypertension, diabetes, hepatitis, tuberculosis, dialysis, Charlson's comorbidity index (CCI) score)

Table S3. Results of logistic regression on the patient satisfaction on attending physician in the hospitalist ward compared to the non-hospitalist ward by region with *p*-value ‡.

	Medical Division			
	Internal Medicine		Surgery	
	OR	p-value	OR	*p*-value
**Accessibility to attending physician**				
I was able to meet attending physician soon after the admission	3.38	<0.0001	1.30	0.2165
I was able to consult with attending physician when I request a consultation	3.59	<0.0001	1.15	0.5576
Attending physician has responded quickly upon the pain management request	3.90	<0.0001	1.06	0.7616
Attending physician has responded quickly upon medicine and procedure request	2.84	<0.0001	1.14	0.4879
I was able to meet attending physician more than twice a day (including rounding)	3.47	<0.0001	3.68	0.0000
Attending physician has spent adequate amount of time in consultation, procedure, and care services	3.19	<0.0001	1.20	0.3881
I was able to have answer to my question related to care during admission period of time	3.09	<0.0001	1.06	0.7784
**Attending physician's consultation and care service**				
Attending physician let me talk without interrupting	2.70	<0.0001	1.19	0.4086
Attending physician checked to be sure I understood everything	2.53	<0.0001	1.15	0.5488
Attending physician communicated fully related to my care and possible negative outcomes	2.01	<0.0001	0.98	0.9321
Attending physician was not in a rush when he/she was with me	2.55	<0.0001	0.82	0.3905
Attending physician's explanation was easy to understand	2.86	<0.0001	1.20	0.4052
Attending physician showed interest in my views and options about my health	2.88	<0.0001	1.55	0.0418
How do you rate attending physician's skill in diagnosing and treating your medical condition?	2.29	<0.0001	0.92	0.7096
Attending physician kept me informed of the plans for my care	2.71	<0.0001	0.89	0.6179
How do you rate attending physician's fund of knowledge?	1.71	0.0001	1.43	0.1014
Attending physician effectively prepared me for discharge	1.77	0.0003	1.08	0.7647
Attending physician re-explained discharge guidelines in details at discharge	2.27	<0.0001	1.03	0.9069
**Surgical patient only**				
I am satisfied with the overall treatment and management after surgery			1.02	0.9361
I am received satisfactory care when I requested for pain control at the surgical site			1.10	0.6860
I am satisfied with the operation site infection management			1.17	0.4974
**Satisfaction evaluation**				
Overall satisfaction on attending physician (beta, *p*-value)	0.610	<.0001	0.253	0.0117
Overall satisfaction on hospital service (beta, *p*-value)	0.554	<.0001	0.138	0.2289

**Overall satisfaction on my health status** prior to the admission (beta, *p*-value)	0.296	<.0001	0.293	0.0156
I would pay extra cost to admitted in the medical ward where care and services are provided specialist (hospitalist)	37.94	<0.0001	29.65	0.0014

‡Fully adjusted for the analysis (adjusted variables: sex, age, medical division, admission type, and region, surgery, general anesthesia, intensive care unit (ICU) transfer, death, hypertension, diabetes, hepatitis, tuberculosis, dialysis, Charlson’s comorbidity index (CCI) score)
